# Identification of Circular RNAs in Hypothalamus of Gilts during the Onset of Puberty

**DOI:** 10.3390/genes12010084

**Published:** 2021-01-12

**Authors:** Qingnan Li, Xiangchun Pan, Nian Li, Wentao Gong, Yaosheng Chen, Xiaolong Yuan

**Affiliations:** 1 State Key Laboratory of Biocontrol, School of Life Sciences, Sun Yat-Sen University, North Third Road, Guangzhou Higher Education Mega Center, Guangzhou 510006, China; liqn27@mail2.sysu.edu.cn; 2 National Engineering Research Center for Breeding Swine Industry, Guangdong Provincial Key Lab of Agro-Animal Genomics and Molecular Breeding, College of Animal Science, South China Agricultural University, Guangzhou 510642, China; 15186255977@126.com (X.P.); 17746076937@163.com (N.L.); g_w_tao@163.com (W.G.); 3 Guangdong Provincial Key Laboratory of Laboratory Animals, Guangdong Laboratory Animals Monitoring Institute, Guangzhou 510260, China

**Keywords:** hypothalamus, puberty, circRNAs, pubertal genes

## Abstract

The disorders of puberty have shown negative outcomes on health of mammals, and the hypothalamus is thought to be the main regulator of puberty by releasing GnRH. Many studies show that the circular RNAs (circRNAs) might be implicated in the timing of puberty in mammals. However, the circRNAs in the hypothalamus of gilts have not been explored. To profile the changes and biological functions of circRNAs in the hypothalamus during the onset of puberty, RNA-seq was utilized to establish pre-, in-, and post-pubertal hypothalamic circRNAs profiles. In this study, the functions of hypothalamic circRNAs were enriched in the signaling pathway of neurotrophin, progesterone-mediated oocyte maturation, oocyte meiosis, insulin, ErbB, and mTOR, which have been highly suggested to be involved in the timing of puberty. Furthermore, 53 circRNAs were identified to be putative hypothalamus-specific expressed circRNAs, and some of them were exclusively expressed in the one of three pubertal stages. Moreover, 22 differentially expressed circRNAs were identified and chosen to construct the circRNA-miRNA-gene network. Moreover, 10 circRNAs were found to be driven by six puberty-related genes (*ESR1*, *NF1*, *APP*, *ENPP2*, *ARNT*, and *DICER1*). Subsequently, the expression changes of several circRNAs were confirmed by RT-qPCR. Collectively, the preliminary results of hypothalamic circRNAs provided useful information for the investigation of the molecular mechanism for the timing of puberty in gilts.

## 1. Introduction

In female pigs, puberty is widely defined as the emergence of the first estrous and capable of reproduction [1]. There are more evidences demonstrated that gilts having an earlier age at puberty can shorten the generation interval of livestock [2,3] and farrow multiple litters [4]. Nevertheless, the basic molecular mechanisms that regulate the onset of puberty have not been largely explored in gilts. Generally, the onset of puberty is controlled and driven by hypothalamic-pituitary-gonadal (HPG) axis. The release of gonadotropin-releasing hormone (GnRH) from the hypothalamus leads to the release of FSH and LH from the pituitary [5], and the FSH and LH act on the folliculogenesis, oogenesis, and sex steroid of the gonads to arouse the timing of puberty in mammals [6]. Lomniczi, A. et al. showed that disrupting the release of pulsatile GnRH in hypothalamus delayed puberty [7]. Pandolfi, E.C. et al. demonstrated that the deletion of homeodomain protein sine oculis-related homeobox 6 (Six6) in hypothalamic GnRH neuron can leads to infertility [8]. These demonstrations indicate that the hypothalamus plays an essential role in the onset of puberty.

Circular RNAs (circRNAs) are covalently closed transcript generated by the back-splicing. This back-splicing jointed a canonical 5′ splice site sequence to an upstream 3′ splice site sequence to produce the only region of a circRNA (BMJ, back-spliced junction) [9]. Multiple circRNAs have been showed to be generated by a single gene through alternative splicing [10]. Recently, next-generation sequencing has shown that circRNAs are widespread expression in mammals [11,12,13]. Moreover, circRNAs have been suggested to be stage-specific, cell- specific, and tissue-specific in the development of mammals [10,14]. Furthermore, most circRNAs are consisted of exons, while a few numbers of circRNAs are formed by the exon-intronic or intronic RNA in mammals [13]. It has been shown that the exonic circRNA may induce active DNA methylation through recruit specific protein, such as the FLI1 exonic circRNA recruits the methylcytosine dioxygenase TET1 to the promoter region of its parental gene [15].

Recently, several studies have showed that circRNAs could regulate the transcription of genes [16,17,18]. Specifically, circRNAs can be used as sponge for microRNAs (miRNAs). For example, Hall et al. showed that circ_Lrp6 was the sponge for circ_Lrp6 to counterbalance functions of the miRNA in functions of the miRNA in VSMCs [19]. Jost et al. produced the artificial circRNAs to inhibit the viral protein production by acting as sponges for the miRNA relevant in human disease [20]. Moreover, increasing evidence has shown that circRNAs are significantly enriched in mammalian brain and are related to physiological development of the brain [21]. Study has shown that the unique patterns of circRNAs across tissues and development stages seemed to reflect the reproductively capable individuals [22]. In addition, recent research indicated that circRNAs were closely connected to development in pig’s brain. For instance, M.T. et al. identified large amounts of circRNAs in fetal brain of pig, and indicated that circRNA was significant impacted gilts’ brain development [23]. These results indicated that circRNAs might play an indispensable role in multiple critical biological process in pigs. However, circRNAs has rarely been studies in onset of puberty of gilts. 

In this study, the hypothalamus of pre-, in-, and post-pubertal gilts were utilized for RNA-seq analysis to explore the expression of circRNAs driven by the pubertal genes, and then investigate a circRNA-miRNA-gene network. It is hoped that the results of this study will provide insight into the potential function of circRNA in gilts during the onset of puberty and help in identifying circRNAs that play pivotal role in this process.

## 2. Materials and Methods

### 2.1. Ethics Statement

All animal experiments were approved by the Animal Care and Use Committee of the South China Agricultural University, Guangzhou, China (permit number: SCAU#2013-10), and conducted with the Regulations for the Administration of Affairs Concerning Experimental Animals (Ministry of Science and Technology, China; revised in June 2004).

### 2.2. Animals

All the experimental Landrace × Yorkshire crossbred gilts were monitored periodically for signs of puberty, including body weight, days of age, change of vulva, and the reaction to the boars, and the onset of puberty was identified by looking at this information. After that, three stages during the onset of puberty (pre-, in-, and post-puberty) were used. Thereinto, three gilts were designated as pre-puberty gilts (160 days old) without any pubertal signs (weight = 81.38 ± 2.40 kg); three gilts were selected as the in-pubertal gilts which exhibited first pubertal signs (weight = 110.00 ± 2.00 kg); three gilts (14 days old) were served as the post-pubertal gilts beyond the pubertal phase (weight = 122.82 ± 9.11 kg). After euthanasia, the hypothalamuses of gilts were immediately removed, placed in the liquid nitrogen, and then stored it at −80 °C until further use. In addition, refer to other researchers’ experimental studies on circRNAs, three replicates in each group were used in this study [24].

### 2.3. RNA Sequencing and the Transcriptome Assembly

Total RNA from the pre-, in- and post-pubertal hypothalamuses of gilts was isolated with the Trizol agent (Invitrogen, Carlsbad, CA, USA). After quality testing of total RNA using the Agilent Bioanalyzer 2100 System (Agilent Technologies, Santa Clara, CA, USA), RNA samples with RNA integrity value of greater than 7.0 were left behind. Subsequently, rRNA was removed using the Epicentre Ribo-zero rRNA removal kits (Epicentre, Madison, WI, USA). Then we used the rRNA-depleted RNAs to compose double-stranded cDNA with the mRNA-Seq Sample Preparation Kit (Illumina, San Diego, CA, USA). Each sample was sequenced using the HiSeq 3000 sequencer according to the manufacturer’s instructions for 5 μg cDNA and generated 150 bp paired-end reads. These raw reads were subjected to quality control using the Cutadapt software [25] to remove the 3′ adaptor-trimming, the low-quality reads which had >10% of unknown bases or >50% of the low-mass bases. Remaining reads after quality control were clean reads, which will be mapped onto the pig reference genome Sus scrofa11.1 by BWA [26] and bowtie2 [27] software. 

### 2.4. circRNA Identification and Data Analysis

CIRI2 [28] was used to identify circRNA after BWA, and find_circ [29] was used to identify circRNAs after bowtie2, which based on the reference genome alignment. We screened the number of unique junctions read to be at least 2, removed RNA with unclear breakpoints, and filtered out RNA with a length greater than 100 kb (genome length) as potential circRNA. Analysis included three replicates for each stage. Finally, circRNAs in pre-, in- and post-pubertal hypothalamuses of gilts were identified. Subsequently, the two software identified the intersection of circRNAs as the candidate circRNAs, and the annotation of circRNAs was proposed by CIRI for further study, which was based on the annotation file from Ensembl release 95. Furthermore, circRNAs originating from exons was used for further analyses. Length of circRNA is the sum of the lengths of the exons that form the circRNA. Besides, we obtained the circRNAs expression with BSJ reads, and used EBSeq package to calculate RPM [30]. In addition, the screening criteria for differential expression were FDR < 0.05, log2 − fold − change −≥1. Furthermore, the screening criteria for stage-specific circRNAs were as follows: circRNA detecting only in a unique stage was judged as stage-specific circRNA. The tissue-specific screening criteria were as follows: identification of circRNAs in this study were matched with the known pig’ circRNAs through the starting and ending genomic positions of circRNAs, and the new circRNAs that were not matched in the database were regarded as the presumed tissue-specific circRNAs. In this study, the ggsignif package was used to perform statistical tests for differences between groups (Welch two-sample *t*-test).

### 2.5. Pathway Analysis and circRNA-miRNA-mRNA Network Construction

The parental genes of circRNA were used for Gene Ontology (GO) analysis and Kyoto Encyclopedia of Genes and Genomes (KEGG) pathway enrichment analysis that the cutoff criterion was *p* < 0.05 and the results were conducted with KOBAS 3.0 online software (http://kobas.cbi.pku.edu.cn/) [31]. In addition, the differentially expressed genes were screened under the condition of FDR < 0.05, log2 − fold − change −≥3. Furthermore, miRanda software [32] was used to predict circRNA-miRNA connections, miRanda match score ≥120. Then miRanda was as well as used to predict differentially expressed target genes of these miRNA, miRanda match score ≥200. Finally, cytoscape software [33] was used to draw a network interaction map between circRNA-miRNA-gene. Moreover, this analysis is based on the part of the transcript containing only exons.

### 2.6. circRNA Validation by RT-qPCR

We used RT and quantitative PCR (RT-qPCR) assays to validate the reliability of the high-throughput RNA sequencing data with divergent primers flanking the BSJ [9]. Used primeScript RT Reagent Kit (TaKaRa, Osaka, Japan) in a Mx3005P real-time PCR System (Stratagene, La Jolla, CA, USA) for qPCR according to the manufacturer’s protocol. Furthermore, the divergent primers of 5 circRNAs were designed to verify the accuracy of the RNA-seq. In order to normalize the expression of circRNAs, GAPDH was served as an internal reference. The PCR standard procedure were denaturation 94 °C (5 min), 40 cycles at 94 °C (10 s), 52 to 62 °C (15 s), and 72 °C (30 s). We used the 2^−ΔΔCt^ method to analyze the RT-qPCR data. The pre-, in- and post-pubertal hypothalamuses were come from three gilts. Moreover, three biological replicates were carried out in each qRT-PCR. The Student’s t test was used to assess the differences between any two pubertal groups of gilts, and the screening criteria for statistically significant were *p* < 0.05. 

## 3. Results

### 3.1. Identification of Hypothalamus-Derivced circRNAs during the Onset of Puberty

**Figure 1:**
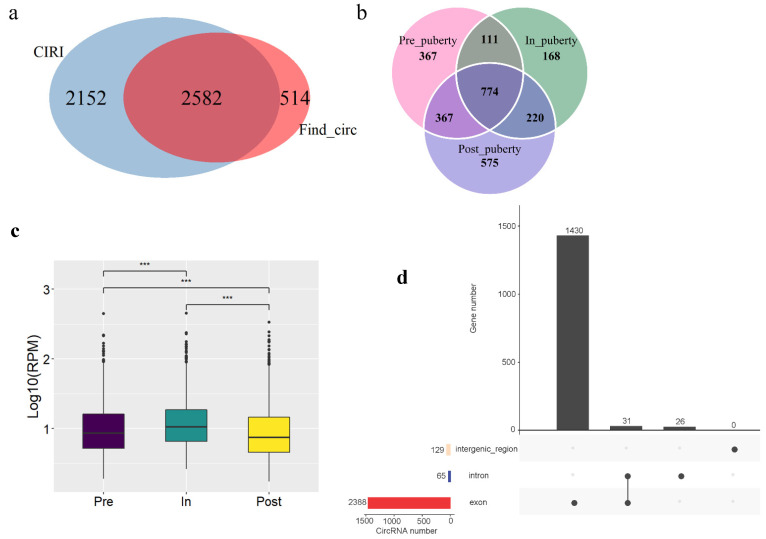
Overview of the identified circRNAs by RNA-seq analyses in ovaries of gilts. (**a**) The circRNAs were identified by two algorithms (CIRI and find_circ). (**b**) The number of unique and common circRNAs during pre-, in- and post-puberty. (**c**) Expression level of circRNAs in the pre-, in- and post-puberty stages. (**d**) The number of three types of circRNA and the number of corresponding parental genes. *** *p* < 0.001.

Totally, 2582 circRNAs candidates were identified by CIRI2 and find_circ software (Figure 1a, Supplementary Table S1). Respectively, 1619, 1273, and 1936 circRNAs were identified during the pre-, in- and post-puberty stages (Figure 1b). And the average circRNAs expression were highest in in-puberty compared with other two stages (Figure 1c). Moreover, circRNAs split into three categories: 2388 exonic circRNAs, 65 intronic circRNAs and 129 intergenic region circRNAs. Furthermore, these 2582 circRNAs were derived from 1487 genes, of which 1461 genes identified as able to produce 2388 exonic circRNA and 57 genes identified as able to produce 65 intronic circRNA (Figure 1d). Furthermore, the 2388 exonic circRNAs were used for subsequent analyses. 

### 3.2. Key Pathways of cirRNAs in Pubertal Transition

**Figure 2:**
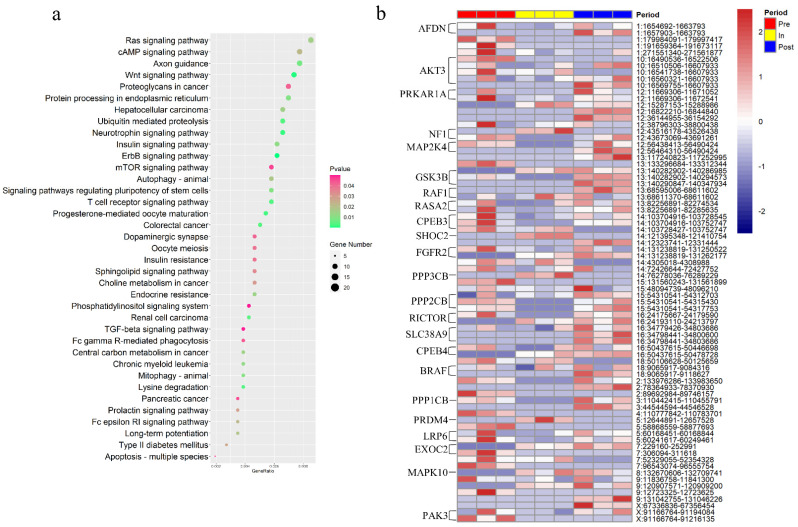
The Key signaling pathway of CirRNAs in pubertal transition. (**a**) KEGG analysis of all identified circRNAs (*p* < 0.05). (**b**) Expression level of circRNAs involved in pubertal key pathways in pre-, in- and post-puberty.

To further explored the circRNA involved in pubertal hypothalamus, the KEGG analysis was used to perform the parental genes of all circRNAs (Supplementary Table S2). Notably, the functional pathways that were significantly overrepresented in pubertal hypothalamus included ras signaling pathway, insulin signaling pathway, ErbB signaling pathway, mTOR signaling pathway, neurotrophin signaling pathway, progesterone-mediated oocyte maturation and oocyte meiosis signaling pathway (Figure 2a). For the ras signaling pathway, *NF1* drive to “circ 12:43516178-43526438” which was uniquely expressed in the in-puberty, and drive to “circ 12:43673069-43691261” which was expressed in pre- and post-puberty; *EXOC2* drive to “circ 7:229160-252991” which was uniquely expressed in in-puberty and post-puberty, and drive to “circ 7:306094-311618” which was expressed in pre-puberty (Figure 2b, Supplementary Table S2). For the insulin signaling pathway, *PPP1CB* drive to “circ 3:110442415-110455791” which was expressed in pre- and post-puberty, but not expressed in post-puberty (Figure 2b, Supplementary Table S2). For the ErbB signaling pathway, *MAP2K4* drive to “circ 12:56438413-56490424” which was expressed in pre- and post-puberty, and drive to “circ 12:56464310-56490424” which was uniquely expressed in post-puberty (Figure 2b, Supplementary Table S2). For the mTOR signaling pathway, *RICTOR* drive to “circ 16:24175667-24179590” which was expressed in pre- and post-puberty, and drive to “circ 16:24193110-24213797” which was expressed in in- and post-puberty (Figure 2b, Supplementary Table S2). For the Neurotrophin signaling pathway, *PRDM4* drive to “circ 5:12644891-12657528” which was uniquely expressed in in-puberty (Figure 2b, Supplementary Table S2). For the progesterone-mediated oocyte maturation, *MAPK10* drive to “circ 8:132670606-132709741” which was expressed in in- and post-puberty, drive to “circ 8:132704592-132733488” which was uniquely expressed in in-puberty (Figure 2b, Supplementary Table S2). For the oocyte meiosis signaling pathway, *PPP3CB* drive to “circ 14:76278036-76289229” which was uniquely expressed in-puberty (Figure 2b, Supplementary Table S2). The detailed information of these circRNA is shown in Supplementary Table S3.

### 3.3. The Stage-Specific circRNAs in the Pubertal Transition

**Figure 3:**
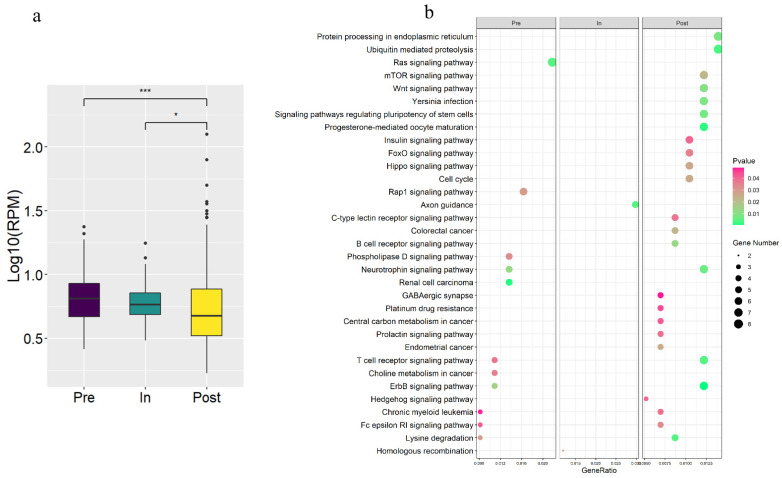
Analysis results of stage-specific circRNAs. (**a**) Expression level of stage-specific circRNAs during Pre-, In-, Post-puberty. (**b**) KEGG analysis with parental genes of stage-specific circRNAs during pre-, in-, post-puberty (*p* < 0.05). * *p* < 0.05, *** *p* < 0.001.

To explore the expression changes of circRNA expressed in all stages, circRNAs were used for differentially expressed analysis except for the stage-specific circRNAs. 367, 168, and 575 putative stage-specific circRNAs were exclusively identified during the pre-, in- and post-puberty stages, respectively (Figure 1b). Furthermore, the expression levels of specific post-puberty circRNAs were significantly lower than specific pre-puberty circRNAs (*t*-test, *p*-value < 9.7 × 10^−5^), as well as significantly lower than specific in-puberty circRNAs (*t*-test, *p*-value < 0.025) (Figure 3a). In addition, the KEGG enrichment with the parental genes of stage-specific circRNAs were showed in Figure 3b, neurotrophin signaling pathway and ErbB signaling pathway were enriched in the pre- and post-puberty stages; axon guidance pathway was enriched in the in-puberty stage; insulin signaling pathway as well as progesterone-mediated oocyte maturation were enriched in the post-puberty stage (Supplementary Table S4). Moreover, 111 genes generated stage-specific and non-specific circRNAs, of which 100 genes generated pre-specific and non-specific circRNAs, and 11 genes generated in-specific and non-specific circRNAs (Supplementary Table S5).

### 3.4. Potentially Regulated Network of Differentially Expressed circRNAs

**Figure 4:**
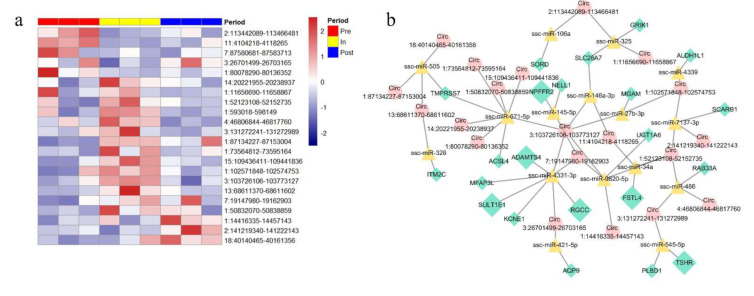
Analysis of differentially regulated circRNAs. (**a**) Heatmap of differentially expressed circRNAs in pubertal transition. (**b**) Differentially expressed circRNAs interact with differentially expressed genes via miRNAs and the differentially regulated status was show in Tables S6 and S7. The red circle represented circRNAs, the yellow triangle represented miRNAs, the green diamond represented genes.

In order to explore the putative functions of differentially expressed circRNAs, we identified a total of 22 differentially expressed circRNAs (Supplementary Table S6) and showed the expression in Figure 4a. Thereinto, 11 differentially up-regulated circRNAs and three differentially down-regulated circRNAs were identified in the pre- vs. in-puberty group; two differentially up-regulated circRNAs and four differentially down-regulated circRNA were identified in the pre- vs. post-puberty group; two differentially up-regulated circRNAs and five differentially down-regulated circRNA were identified in the in- vs. post-puberty group (Supplementary Table S6). Later, circRNAs mentioned above were used to predict the binging sites, and the top three possible miRNA targets were listed in Table 1. After that, differentially expressed genes were used to predict the circRNA-miRNA-gene regulatory network (Figure 4b). Noticeably, we highlight *FSTL4*, *TSHR*, *SULT1E1*, *NPFFR2*, *RGCC*, and *ADAMTS4* genes, which were associated with puberty [34,35,36,37,38,39] (Supplementary Table S7). Interestingly, one of these differentially expressed circRNAs, “circ 11:4104218-4118265” that interacted with *FSTL4* via ssc-miR-34a, was down-regulated in the pre- vs. in-puberty groups, as well as down-regulated in the pre- vs. post-puberty groups (Supplementary Tables S6 and S7). In addition, “circ 3:103726106-103773127” was up-regulated in the pre- vs. in-puberty groups but down-regulated in the in- vs. post-puberty groups (Supplementary Table S6), and this circRNAs interacted with *SULT1E1* via ssc-miR-4331-3p (Supplementary Table S7). According to this result, we found that some differential expression of circRNAs interacted with differentially expressed genes via miRNAs, whereafter they potentially regulate the onset of puberty.

### 3.5. The Hypothalamus-Specific circRNAs in Puberty

**Figure 5:**
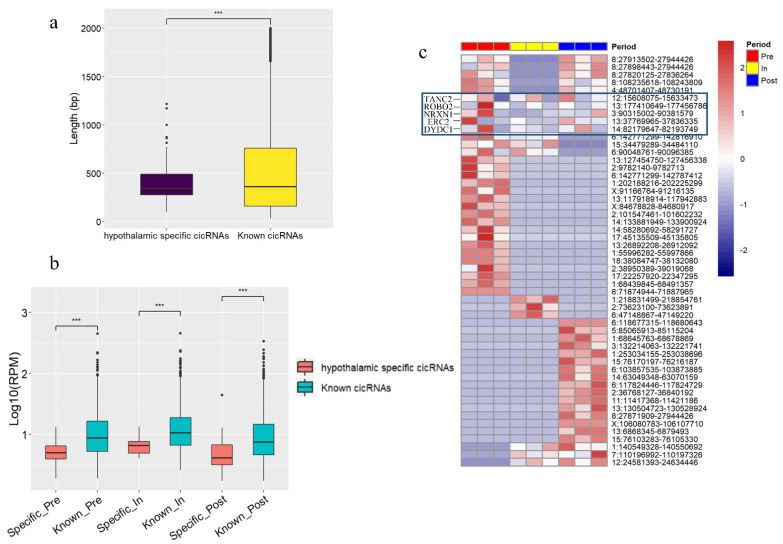
Analysis results of hypothalamus-specific circRNAs. (**a**) Length of tissue-specific circRNAs and known circRNAs. (**b**) Significant difference analysis between hypothalamus-specific circRNAs and known circRNAs. (**c**) The expression of hypothylamus-specific circRNAs in three stages, the blue box represents circRNAs and its parental genes without variation. *** *p* < 0.001.

The known circRNA of pig come from circAtlas 2.0, which was includes thousands of known circRNAs in nine porcine tissue types (brain, heart, kidney, liver, lung, skeletal muscle, spleen, testis, and retina) [40]. In order to investigate the specific circRNAs in hypothalamus tissue, 2518 circRNAs which overlapped in known circAtlas 2.0 were excluded, and leaving another 53 circRNAs as being putative hypothalamus-specific circRNAs. Moreover, the 53 putative hypothalamus-specific circRNAs were significantly shorter than that of the known circRNAs (*t*-test, *p*-value = 0.00082) (Figure 5a). Furthermore, the expression of these putative hypothalamus-specific circRNAs was significantly lower than that of the known circRNAs during the onset of puberty (*t*-test, *p*-value < 0.001) (Figure 5b). In addition, the expression of these 53 hypothalamus-specific circRNAs was shown in Figure 5c, some of which were only expressed in one of three stages, and 5 genes were able to produce circRNAs at all stages without variation. Interestingly, the circRNA “circ 1:68439845-68491357” was only expressed in pre-puberty and “circ 1:68645763-68678869” was only expressed in post-puberty (Supplementary Table S8), both of which were derived from *GRIK2* associated with excitatory neurotransmission in the mammalian central nervous system [41]. The parental genes of these putative hypothalamus-specific circRNAs were enriched in “ssc04360: axon guidance” and “ssc04015: rap1 signaling pathway” pathways (Supplementary Table S9**)**. Meanwhile, these parental genes of hypothalamus-specific circRNAs were related to “GO 0048666: neuron development” and “GO 0030182 neuron differentiation” terms (Supplementary Table S9).

### 3.6. circRNAs in Pubertal Genes

To further explore the function of circRNAs in puberty, the 20 pubertal genes were selected and investigated through reviewing the literature and databases by hand (Supplementary Table S10). Subsequently, we found that 16 circRNAs were driven by 6 pubertal genes (Supplementary Table S10). Thereinto, *ESR1* drive to circRNAs “circ 1:14416335-14457143”, which was differentially expressed during pubertal hypothalamus; *APP* drive to four circRNAs (“circ 13:189505179-189528484”, “circ13:189505179-189544139”, “circ 13:189523366-189528484” and “circ 13:189597995-189600156”); *NF1* drive to two circRNAs (“circ 12:43516178-43526438” and “circ 12:43673069-43691261”); *ENPP2* and *ARNT* respectively drive to circRNAs “circ 4:19360870-19367922” and circRNAs “4:98369520-98372553”, which were uniquely expressed in pre-pubertal hypothalamus; *DICER1* drive to circRNAs “circ 1:14416335-14457143”, which was always expressed during pubertal hypothalamus (Supplementary Table S10). These results will become the focus for further analysis.

### 3.7. Validation of circRNAs by RT-qPCR

**Figure 6:**
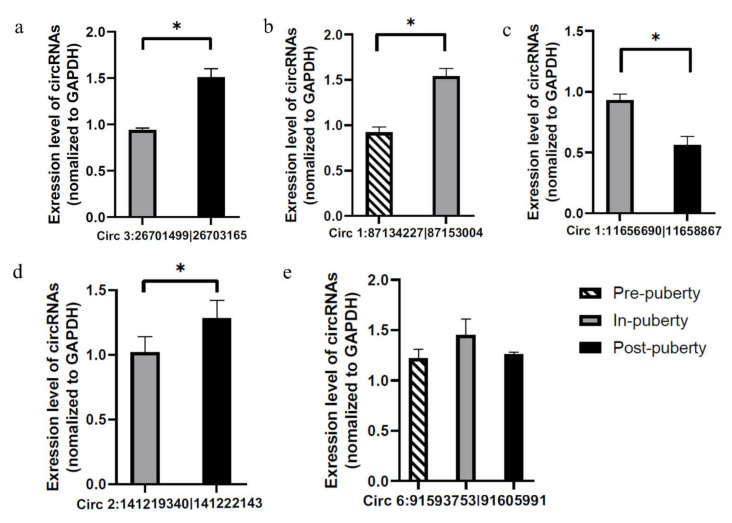
RT-qPCR validation of circRNAs. Five circRNAs were randomly selected for RT-qPCR validation, of which four circRNAs (**a**–**d**) were differential expression and one circRNA (**e**) was no significant difference. The primer informations were listed in Table S11. * *p* < 0.05.

In order to verify the accuracy of RNA-seq data, five circRNAs were randomly selected for validation experiments. Thereinto, circRNA “circ 1:11656690-11658857”, “circ 1:87134227-87153004”, “circ 2:141219340-141222143”, “circ 3:26701499-87153004” were differential expression and circRNA “circ 6:9159375-91605991” was no differential expression. First, the divergent primers were used to this study, then the RT-qPCR were used to verified (see Section 2 for detail). Accordingly, the RT-qPCR assay results showed a similar tendency of expression with our RNA-seq data (Figure 6), further confirming the reliability of sequencing.

## 4. Discussion

Puberty is a complex physiological process regulated by multiple pathways. Hypothalamus, the main female puberty organs, directly mediate the pulsatile release of GnRH, which play crucial roles during onset of puberty [7,42]. Due to the delay in puberty, about 30% of gilts has been culled, which has obviously harmed the financial stake of the modern commercial farms [43]. CircRNAs were found to be function in many biological processes and widely expressed in mammal [44,45]. With the development of next-generation sequencing technology, research on the regulation of circRNA in animal puberty has made small progress year by year. However, puberty-associated circRNA expression remains unclear in gilts. Thus, our study focused on exploring the potential role of circRNAs in pubertal hypothalamus of gilts. A total of 2582 circRNAs were identified, of which 1110 were putative stage-specific circRNAs, 53 were putative hypothalamus-specific circRNAs and 22 were differentially expressed circRNAs. 

For all identified circRNAs, 2388 exonic circRNAs were generated from 1461 parental genes (Figure 1d). This result may be explained by the fact that one gene could produce different circRNAs through different splicing forms. Furthermore, we uncovered several genes in some of the key pathways associated with the timing of puberty that could drive expression of differential circRNAs in differential pubertal stage. Neurofibromin 1 (*NF1*) is the main Ras regulator and plays an important role in neurons [46]. For the ras signaling pathway, “circ 12:43516178-43526438” driven by *NF1* was only expressed in in-puberty, while “circ 12:43516178-43526438”, driven by *NF1*, was not expressed in in-puberty but expressed in pre- and post-puberty, indicating that these two *NF1*-driven circRNAs have different roles during the onset of puberty. When entering in-puberty, *NF1* might specifically splicing “circ 12:43516178-43526438” to play a crucial role. *MAP2K4* as the direct upstream activator of NH2-terminal kinase pathway, which plays an important role in regulating neuron survival and apoptosis in response to cerebral ischemia [47,48]. Similarly, for the ErbB signaling pathway, the two circRNAs driven by *MAP2K4* might have different effects. When entering pre-puberty, *MAP2K4* spliced “circ 12:56438413-56490424”, and after in-puberty, *MAP2K4* re-spliced “circ 12:56438413-56490424” and spliced “circ 12:56464310-56490424”. In addition, previous report has shown that *MAPK10* could block the hypothalamic-pituitary-thyroid axis, thereby reducing energy expenditure and promoting obesity [49]. In this study, *MAPK10* drive to the specific expression of circRNA “circ 8:132670606-132709741” during in- and post-puberty in the progesterone-mediated oocyte maturation signaling pathway. These results suggested that the circRNAs identified on the relevant signaling pathway might play a crucial role in pubertal transition.

Moreover, 367, 168, and 575 circRNAs were uniquely expressed in pre-, in-, post-puberty, respectively (Figure 3b), among which the uniquely expressed circRNAs in post-puberty had the highest average expression (Figure 3a). Importantly, parental genes of stage-specific circRNAs were involved in neurotrophin signaling pathway, ErbB signaling pathway, axon guidance pathway, insulin signaling pathway as well as progesterone-mediated oocyte maturation; these processed have been reported to regulate the puberty [50,51,52,53,54,55]. In addition, circRNA-miRNA-gene network was used to predict the relationship between differential circRNA and differential genes. Interestingly, differentially expressed circRNAs “circ 11:4104218-4118265” that downregulated in the pre- vs. post-puberty groups interacted with *FSTL4* that downregulated in the pre- vs. post-puberty groups (Tables S6 and S7). This result suggested that circRNAs “circ 11:4104218-4118265” might be the sponge for ssc-miR-34, thereby promote the expression of *FSTL4*. The formation of circRNA is affected by alternative splicing and methylation [9]. Moreover, our previous studies have found that there had differential methylation pattern in genes during the onset of puberty in gilts [56]. It is possible, therefore, that the parental genes might be influenced by other epigenetic regulation to produce stage-specific exonic circRNA.

In addition, the circRNA “circ 7:19147980-19162903” that upregulated in the pre- vs. in-puberty groups interacted with *SULT1E1* and *NPFFR2* that up-regulated in the pre- vs. in-puberty groups (Supplementary Tables S6 and S7). This result suggested that circRNAs “circ 7:19147980-19162903” might be the sponge for ssc-miR-4331-3p and ssc-miR-145-5p, thereby promote the expression of *SULT1E1* and *NPFFR2*, respectively. These results showed that there might be a competitive binding of miRNA by circRNA to affect gene expression in pubertal hypothalamus of gilts. Subsequently, we identified 53 hypothetical hypothalamic-specific circRNAs, which were involved in axon guidance and rap1 signaling pathway pathways, neuron development and neuron differentiation. Previous study reported that there were complex changes in the central nervous system in the pubertal hypothalamus [57]. Other study showed that proper axon guidance is essential for the migration of GnRH neurons in the brain [53]. Another study demonstrated that the rap1 plays a crucial role in mediating cAMP-induced MAPK activation of specific cell types [58]. It may be the case therefore that hypothalamus-specific circRNAs affect the development of neurons in hypothalamus and subsequently affect the onset of puberty. Interestingly, we found that two tissue-specific circRNA (circ 1:68439845-68491357, circ 1:68645763-68678869) were derived from the gene associated with excitatory neurotransmission in the mammalian central nervous system, suggesting that these two circRNAs might play a vital role in the specific differentiation of the hypothalamus.

Moreover, 10 pubertal genes-driven circRNAs were found in this study (Supplementary Table S10). Thereinto, *APP*, which implicated in neural development and reproduction [59], drive to four circRNAs (“circ 13:189505179-189528484”, “circ 13:189505179-189544139”, “circ 13:189523366-189528484” and “circ 13:189597995-189600156”); *ESR1*, which associated with the timing of puberty [60], drive to circRNAs “circ 1:14416335-14457143”, of which “circ 1:14416335-14457143” was up-regulated in pre- vs. post-puberty group (Supplementary Table S6). Moreover, *DICER1*, which was essential for the normal development of the reproductive system [61], drive to circRNA “circ 7:116399251-116408577”. In addition, these circRNAs was constituted in multiple exons except “circ 4:47041713-47042593” and “circ 7:116399251-116408577”. Our results provide new insight into the existence of hypothalamus-derived circRNAs in gilts. However, the underlying mechanism of these circRNAs during gilts’ pubertal onset still requires carefully elucidation and verification.

## 5. Conclusions

During pubertal transition, 2582 circRNAs were identified hypothalamus, of which 1110 circRNAs were putative stagce-specific circRNAs, 53 circRNAs were putative hypothalamus-specific expressed circRNAs, and 22 circRNAs were significantly differentially expressed. These cirRNA were mostly enriched in neurotrophin signaling pathway, progesterone-mediated oocyte maturation, ras signaling pathway, insulin signaling pathway, ErbB signaling pathway, mTOR signaling pathway and oocyte meiosis signaling pathway, which had been highly implicated in puberty. Moreover, 16 circRNAs were driven by six genes, i.e., *ESR1*, *NF1*, *APP*, *ENPP2*, *ARNT*, and *DICER1*. These preliminary results indicated circRNAs involved in the timing of puberty at the hypothalamus level in gilts, and provided useful information for the investigation of the molecular mechanism of pubertal onset in mammals.

## Figures and Tables

**Table 1:** The differentially regulated circRNAs in this study of hypothalamus of gilts.

circRNA ID	Position	Strand	circRNA Type	Parental Gene	Top 3 miRNA Targets
1:102571848-102574753	Chr1:102571848-102574753	+	exon	*DCC*	ssc-miR-9814-3p, ssc-miR-15b, ssc-miR-144
1:11656690-11658867	Chr1:11656690-11658867	−	exon	*TIAM2*	ssc-miR-383, ssc-miR-7857-3p, ssc-miR-185
1:14416335-14457143	Chr1:14416335-14457143	−	exon	*ESR1*	ssc-miR-4331-3p, ssc-miR-16, ssc-miR-7143-3p
1:50832070-50838859	Chr1:50832070-50838859	+	exon	*SMAP1*	ssc-miR-4331-3p, ssc-miR-9825-5p, ssc-miR-383
1:52123108-52152735	Chr1:52123108-52152735	+	exon	*RIMS1*	ssc-miR-4331-3p, ssc-miR-574-5p, ssc-miR-34a
1:593018-598149	Chr1:593018-598149	+	exon	*WDR27*	ssc-miR-4331-3p, ssc-miR-7135-3p, ssc-miR-181b
1:73564812-73595164	Chr1:73564812-73595164	+	exon	*SOBP*	ssc-miR-4331-3p, ssc-miR-27b-5p, ssc-miR-491
1:80078290-80136352	Chr1:80078290-80136352	−	exon	*HS3ST5*	ssc-miR-4331-3p, ssc-miR-9820-5p, ssc-miR-9-1
1:87134227-87153004	Chr1:87134227-87153004	+	exon	*PHIP*	ssc-miR-545-3p, ssc-miR-4331-3p, ssc-miR-148b-5p
11:4104218-4118265	Chr11:4104218-4118265	+	exon	*CDK8*	ssc-miR-4331-3p, ssc-miR-9822-3p, ssc-miR-424-5p
13:68611370-68611602	Chr13:68611370-68611602	−	exon	*RAF1*	ssc-miR-208b
14:20221955-20238937	Chr14:20221955-20238937	+	exon	*NEK1*	ssc-miR-4331-3p, ssc-miR-505, ssc-miR-2483
15:109436411-109441836	Chr15:109436411-109441836	−	exon	*NDUFS1*	ssc-miR-30a-3p, ssc-miR-30e-3p, ssc-miR-676-5p
18:40140465-40161356	Chr18:40140465-40161356	−	exon	*BBS9*	ssc-miR-199b-5p, ssc-miR-186-5p, ssc-miR-186-3p
2:113442089-113466481	Chr2:113442089-113466481	−	exon	*FBXL17*	ssc-miR-4331-3p, ssc-miR-145-5p, ssc-miR-320
2:141219340-141222143	Chr2:141219340-141222143	+	exon	*MATR3*	ssc-miR-10390, ssc-miR-186-5p, ssc-miR-421-5p
3:103726106-103773127	Chr3:103726106-103773127	−	exon	*CRIM1*	ssc-miR-574-5p, ssc-miR-4331-3p, ssc-miR-146a-3p
3:131272241-131272989	Chr3:131272241-131272989	+	exon	*RNASEH1*	ssc-miR-149
3:26701499-26703165	Chr3:26701499-26703165	+	exon	*SMG1*	ssc-miR-4331-3p, ssc-miR-106a, ssc-miR-20a-5p
4:46806844-46817760	Chr4:46806844-46817760	+	exon	*NBN*	ssc-miR-4331-3p, ssc-miR-9820-5p, ssc-miR-7140-3p

+ Positive strand; − Negative strand.

## Data Availability

The datasets used in this study have been submitted to the European Nucleotide Archive under accession number PRJEB39729.

## Supplementary Materials

The following are available online at https://www.mdpi.com/2073-4425/12/1/84/s1, Table S1: Information of all identified circRNAs. Table S2: The KEGG pathways enriched using parental genes of all CircRNAs. Table S3: Key pathways related to the timing of puberty in parental genes of circRNAs. Table S4: The KEGG pathways enriched using parental genes of stage-specific CircRNAs. Table S5: Parental Genes That Are Capable of Producing Stage-specific And Non-specific CircRNAss. Table S6: The differentially regulated circRNAs. Table S7: The differentially expressed genes associated with puberty our development of ovary. Table S8: Tissue-specific CircRNAs. Table S9: The KEGG pathways and GO term enriched using parental genes of tissue-specific CircRNAs. Table S10: CircRNAs in Pubertal Genes. Table S11: Primers used for qRT-PCR.
